# Estimating bite force in extinct dinosaurs using phylogenetically predicted physiological cross-sectional areas of jaw adductor muscles

**DOI:** 10.7717/peerj.13731

**Published:** 2022-07-12

**Authors:** Manabu Sakamoto

**Affiliations:** Department of Life Sciences, University of Lincoln, Lincoln, United Kingdom

**Keywords:** Bite force, Dinosaurs, Phylogenetic comparative methods, Phylogenetic predictive modelling, Physiological cross-sectional area, Biomechanics

## Abstract

I present a Bayesian phylogenetic predictive modelling (PPM) framework that allows the prediction of muscle parameters (physiological cross-sectional area, *A*_Phys_) in extinct archosaurs from skull width (*W*_Sk_) and phylogeny. This approach is robust to phylogenetic uncertainty and highly versatile given its ability to base predictions on simple, readily available predictor variables. The PPM presented here has high prediction accuracy (up to 95%), with downstream biomechanical modelling yielding bite force estimates that are in line with previous estimates based on muscle parameters from reconstructed muscles. This approach does not replace muscle reconstructions but one that provides a powerful means to predict *A*_Phys_ from skull geometry and phylogeny to the same level of accuracy as that measured from reconstructed muscles in species for which soft tissue data are unavailable or difficult to obtain.

## Introduction

Biomechanical modelling is an important means to infer the functional performances, ecologies, and behaviours of extinct animals for which such features cannot be directly observed (Anderson et al., 2012), *e.g*., in dinosaurs (Rayfield et al., 2001; Lautenschlager, 2013; Sakamoto, 2010; Gignac & Erickson, 2017; Bates & Falkingham, 2012). Biomechanical modelling can be particularly informative in terms of adaptive evolution and patterns of natural selection, when it outputs a univariate performance measure, such as bite force (Sakamoto, Ruta & Venditti, 2019). This is because performance measures like bite force represent tangible physical interactions with the environment in which the animals live or lived in. Bite force has repeatedly been reported as being correlated with dietary ecology in extant species (Dumont et al., 2012; Santana, Dumont & Davis, 2010; Santana, Strait & Dumont, 2011; Herrel et al., 2005a; Herrel et al., 2009), and thus has been treated as being likely informative for extinct species.

As bite force is the output of a musculo-skeletal lever system (Sinclair & Alexander, 1987), its estimation relies on input parameters including skeletal morphology and those derived from muscle anatomy and architecture, the latter of which is seldom preserved in fossils. Muscles thus need to be reconstructed first, then relevant muscle parameters estimated (Rayfield et al., 2001; Gignac & Erickson, 2017; Bates & Falkingham, 2012; Mazzetta, Cisilino & Blanco, 2004; Mazzetta et al., 2009). These parameters include the positions and orientations of muscle bodies, the weight, volume, and density of the muscle bodies, lengths of the muscle fibres, and the pennation angles of muscle fibres, all of which contribute to the physiological cross-sectional area (*A*_Phys_) of muscles and capacity to generate force. However, muscle parameters based on reconstructions are associated with some degree of uncertainty (Gignac & Erickson, 2017; Bates & Falkingham, 2018). This owes to a number of reasons but chief among them is the unknowability of fiber lengths and pennation angles in fossil species. These parameters vary substantially amongst living species and are generally poorly documented. Fibre lengths and pennation angles (but especially the former) are crucial architectural data in estimating *A*_Phys_, which itself being the determining factor of bite force. This is because force is proportional to *A*_Phys_ (Sinclair & Alexander, 1987) and thus the latter can be used to estimate isometric muscle force using a known stress factor σ (commonly 0.3 N/mm^2^).

Owing to difficulties and challenges facing muscle parameter reconstructions combined with the impact it has over downstream biomechanical modelling, there is need for a simple but reasonably accurate method of predicting *A*_Phys_ from skull geometries. Here, I present a Bayesian predictive modelling framework, the phylogenetic predictive model (PPM) (Organ et al., 2007; Sakamoto, 2021), to generate posterior predictive distributions of *A*_Phys_ from relationships between *A*_Phys_ and a skull geometry predictor variable, the skull width (*W*_Sk_). Crucially, the aim of this article is not to present a method that accurately predicts *A*_Phys_ in fossil species from skull geometries as a substitute of muscle reconstruction, but a method that can predict *A*_Phys_ from skull geometries and phylogeny to the same level of accuracy as that measured from reconstructed muscles. Thus, the main objective is to provide the community with a tool to aid in reasonably accurate estimates of *A*_Phys_ in fossil organisms for which muscles are difficult to reconstruct.

## Materials and Methods

### Functional muscle groups

**Figure 1:**
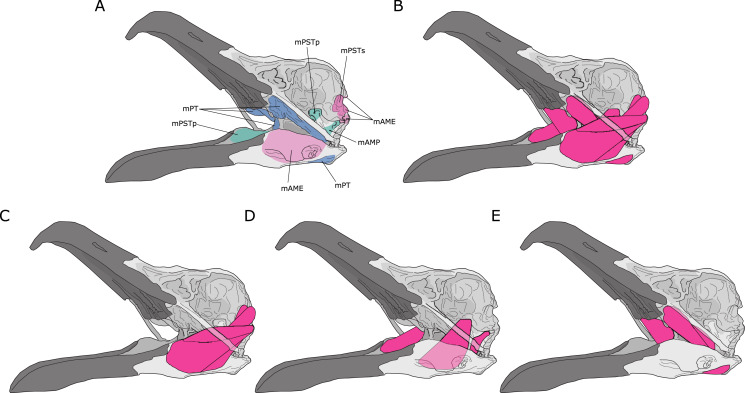
Jaw adductor muscles and functional muscle groupings in extant archosaurs. (A) Attachment sites for jaw adductor muscles are depicted on a skull of a herring gull (*Larus fuscus*). Abbreviations are as follows: mAME, M. adductor mandibulae externus; mPSTs, M. pseudotemporalis superficialis; mPSTp, M. pseudotemporalis profundus; mAMP, M. adductor mandibulae posterior; and mPT, M. pterygoideus. Adductor muscle anatomy is then depicted for: (B) all adductor muscles; (C) temporal muscle group (mAME + mPSTs); (D) the quadrate muscle group (mPSTp + mAMP); and (E) the pterygoid muscle group (mPT).

Jaw adductor muscles in archosaurs are largely grouped and named based on developmental biology and various topological criteria such as their relative positioning to nerves and blood vessels (Holliday & Witmer, 2007). However, from a functional perspective, the existing groupings are not necessarily congruent with lines of actions in a lever model. For instance, the *Musculus* (M) *pseudotemporalis superficialis* (mPSTs) is topographically and functionally similar to the *M. adductor mandibularis externus* (mAME) but are developmentally linked to the *M. pseudotemporalis profundus* (mPSTp), the latter of which is often physically connected to (and indistinguishable from) the *M. adductor posterior* (mAMP). This largely stems from the fact that the mPSTs and mAME both have cranial attachments in the temporal fossa, while the mPSTp and mAMP both attach onto the quadrate (Fig. 1). Thus, the mPSTs and mAME work together as inter-linked functional in-levers while the mPSTp and the mAMP work together as a separate set of inter-linked functional in-levers.

I distinguish three functional adductor groupings, largely following Rayfield et al. (2001) and Sakamoto (2010), and identified muscle body complexes as follows: (1) the temporal muscle group (mTemp), consisting of mAME and mPSTs; (2) the quadrate muscle group (mQuad), consisting of the mPSTp and mAMP; and (3) the pterygoid muscle group (mPt), consisting of the M. pterygoideus (mPT). Practically, these approximate groupings are necessary as adductor muscles in smaller specimens are often difficult, if not impossible, to separate into the classic topological groupings, and as the goal of this study is to predict *A*_Phys_ in fossil species where we do not necessarily have detailed topological information. Furthermore, in the context of both biomechanical modelling and predictive modelling, approximation is often important in obtaining *accurate* predictions, which is not necessarily the most *precise* model.

### Physiological cross-sectional areas in extant species

*A*_Phys_ for extant species (*N* = 39) were calculated from muscle architecture data collected predominantly from the literature but also from dissections (Sakamoto, Ruta & Venditti, 2019) (*Struthio camelus*, one specimen; *Buteo buteo*, three specimens; *Larus fuscus*, two specimens; *Branta canadensis*, one specimen; *Gallus domesticus*, two specimens; File S1; specimens were collected by the Bristol Ornithological Club and were donated to the University of Bristol as part of a clinical veterinary anatomy lab practical, c. 2005–2006 (Sakamoto, 2008)). *A*_Phys_ were calculated as:

(1) $${A_{\rm Phys}} = {(M{\rm cos \theta}) / (\rho L),}$$

following Sacks & Roy (1982), where *M* is the wet weight of the muscle body (g), θ is the mean pennation angle, ρ is the specific density (1.056 × 10^−3^ g/mm^3^ (Burkholder et al., 1994)), and *L* (mm) is mean fiber length. In the case of parallel fibers θ is 0° and thus cosθ is 1.

Muscle measurements for *A*_Phys_ calculations were taken for two specimens of *Buteo buteo* and one specimen each of *Larus fuscus* and *Struthio camelus*. Muscles were weighed prior to sectioning to obtain *M*. Muscles were carefully sectioned under a microscope using a sharp scalpel. Incisions were made parallel to the length of the muscle fibers as much as possible. *L* and θ were measured using ImageJ (Rasband, 2012). As sectioning muscles can artificially truncate fibers and ends of fibers can often be obscured and difficult to determine, fiber lengths were taken at multiple locations on one or more sections through each muscle, the mean of which was taken as *L*.

In some specimens, *A*_Phys_ were approximated using the gross cross-sectional area (*A*_Gross_), as simply the cross-section taken perpendicular to the long axis of the muscle body (Rayfield et al., 2001). *A*_Gross_ was measured in one specimen each of *S. camelus*, *B. buteo*, and *Branta canadensis*, and two specimens each of *L. fuscus*, and *Gallus gallus*. The muscle body was sectioned roughly perpendicular to the major axis of the muscle body at its widest point, and its *A*_Gross_ was digitally measured using Image J. The mean value of the left and right sides was taken as the final *A*_Gross_ value. Comparisons between *A*_Gross_ measurements and *A*_Phys_ calculations (following Eq. (1)) taken from different specimens within the same species, reveal that measured *A*_Gross_ values are generally congruent with calculated *A*_Phys_ values (Sakamoto, 2008; Persons & Currie, 2011; Snively & Russell, 2007).

### Physiological cross-sectional areas in extinct species

For extinct archosaurs, cross-sectional areas of the jaw adductor muscles were estimated as *A*_Gross_ using a variant of the dry skull method (Thomason, 1991), whereby cranio-mandibular dimensions (namely the areas of the supratemporal, subtemporal, and mandibular fenestrae) were used to bound the *A*_Gross_ of individual jaw adductor muscles. I measured *A*_Gross_ of the mAME, mPSTs, mPSTp + mAMP and mPT on photographs and diagrams of reconstructed skulls taken at various angles of view. These are conceptually similar to previously published methods to estimate *A*_Gross_ in extinct dinosaurs (Rayfield et al., 2001; Mazzetta et al., 2009; Reichel, 2010). I further applied muscle pennation angle θ = 45° for the mTemp group, θ = 0° for the mQuad group, and θ = 30° for the mPt group, based on average pennation angles in my extant archosaur samples. I applied the effects of pennation on to *A*_Gross_ through division of *A*_Gross_ by sinθ. This approximates *A*_Phys_ in fossil archosaurs.

### Predictor variable

**Figure 2:**
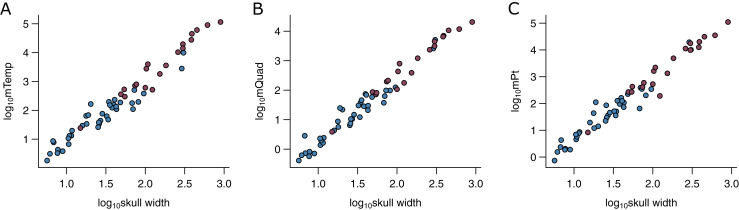
Relationships between physiological cross-sectional areas and skull width in the PPM training set (*N* = 59). Relationships between physiological cross-sectional areas *A*_Phys_ for each of the three muscle groups and skull width (*W*_Sk_) are shown for extant (blue) and extinct (red) archosaurs in the PPM training set (*N* = 59): (A) temporal muscle group (mTemp); (B) quadrate muscle group (mQuad); and (C) pterygoid muscle group (mPt).

I used the width of the skull (*W*_Sk_) as the predictor variable in the phylogenetic predictive models (PPMs). *W*_Sk_ was chosen here as it has previously been demonstrated to predict bite force (Herrel et al., 2005a; Sakamoto, 2021; Herrel et al., 2005b) and various jaw adductor muscles well (Fig. 2). It is also readily available from the literature and easy to measure for a vast number of species for which muscle data do not exist, both extant and extinct. The utility of its wider applicability makes simple measures like *W*_Sk_ an ideal predictor in predictive modelling. *W*_Sk_ were mostly measured directly from osteological and fossil specimens where possible but augmented with data taken from photographs and literature.

### Phylogeny

**Figure 3:**
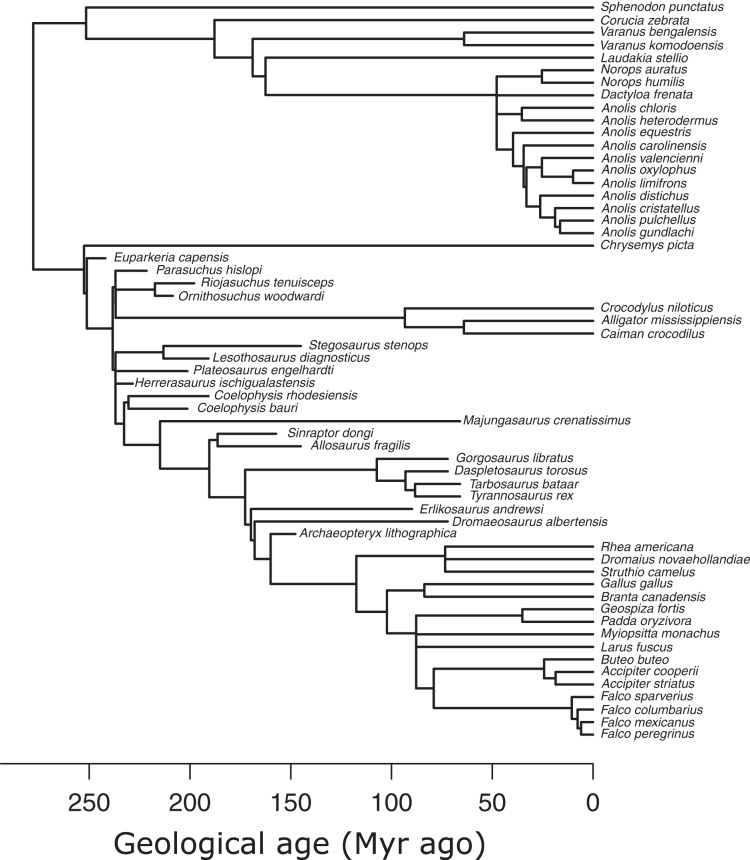
Phylogeny of extant and extinct saurians (*N* = 59) used in the phylogenetic predictive modelling. The extant portion of the tree was taken from the TimeTree of Life and extinct tips inserted at the relevant positions.

I used an informal supertree of saurians based on the Time Tree of Life (TTOL) (Kumar et al., 2017) with fossil tips inserted manually at the appropriate phylogenetic locations (Sakamoto, Ruta & Venditti, 2019) (Fig. 3). Divergence times for fossil branches are based on first appearance dates (FAD) with terminal tips extended to their last appearance dates (LAD) using the paleotree R package (Bapst, 2012). I used the full range of temporal durations to scale the branches, as this allows for the maximum amount of time possible for trait evolution to occur (Sakamoto, Ruta & Venditti, 2019). Zero-length internal branch lengths were resolved by sharing time with neighbouring branches using the “equal” method (Bapst, 2012; Brusatte et al., 2008). While there are alternative methods to scaling branches, *e.g*., tip-dating using the fossilised birth-death model (Drummond & Stadler, 2016), phylogenetic regression under Brownian motion, which underlies the PPM framework, is extremely robust to uncertainties in branch lengths (Stone, 2011), so the choice of branch scaling makes minimal impact on PPM.

### Phylogenetic predictive modelling

I used a Bayesian PPM (Organ et al., 2007; Sakamoto, 2021) to predict *A*_Phys_ in extinct archosaurs. Separate PPMs were fitted on each of the three muscle groups as outlined above with the relevant *A*_Phys_ as the response variable and *W*_Sk_ as the predictor variable.

Model performance, or prediction accuracy, of each PPM was evaluated in a dataset containing only the extant species (*N* = 39) first, through Leave-One-Out Cross-Validation (LOOCV). LOOCV procedure largely follows that outlined in (Sakamoto, 2021), and is as follows: (1) the PPM was first fit on the dataset leaving one species out (N − 1) using Markov Chain Monte Carlo (MCMC) generating a posterior distribution of predictive models; (2) the posterior predictive models were used to predict *A*_Phys_ for the species that was left out from Step 1 based on the *W*_Sk_ and phylogenetic position of that species; (3) the posterior distribution of predictions (posterior predictive distribution) was evaluated against the actual *A*_Phys_ value recorded for that species. If the observed value fell outside the vast majority of the posterior predictive distribution (*i.e*., beyond 95% of the distribution; *p*_MCMC_ < 0.05), then it is deemed that the actual *A*_Phys_ value is significantly different from the posterior predictive distribution, meaning that the prediction has failed in this particular species. I repeated these steps for all species in the data set (*N* = 39) and calculated the proportion of species for which the model succeeded in accurately predicting *A*_Phys_ out of the total sample size *N*.

I then predicted *A*_Phys_ for 53 fossil species of archosaurs (predominantly theropod dinosaurs). I first fitted a PPM on the *N* = 39 dataset and generated a posterior distribution of predictive models. I then used the predictive models to generate posterior predictive distributions for all 53 fossil species using their *W*_Sk_ and phylogenetic positions. This procedure is largely identical to LOOCV but is conducted in one step instead of one species at a time (Sakamoto, 2021). For 20 of the 53 fossil species, *A*_Phys_ measured from reconstructed muscles exist, thus allowing for assessment of the match between PPMs-predicted *A*_Phys_ and reconstructed *A*_Phys_ in fossil species.

Additionally, I evaluated prediction accuracy of PPMs on an expanded training set (*N* = 59) that includes *A*_Phys_ for select fossil species (*N* = 20) measured from reconstructed muscles (Sakamoto, Ruta & Venditti, 2019) or taken from literature (Lautenschlager, 2013; Gignac & Erickson, 2017; Bates & Falkingham, 2012; Lautenschlager et al., 2016). Prediction accuracy was evaluated through LOOCV as outlined above.

I then predicted *A*_Phys_ for the remaining 33 fossil species of dinosaurs (predominantly theropods). I first fitted a PPM on the *N* = 59 dataset and generated a posterior distribution of predictive models. I then used the predictive models to generate posterior predictive distributions of *A*_Phys_ for all 33 fossil species using their *W*_Sk_ and phylogenetic positions.

All model fitting was conducted in BayesTraits V3 over three independent MCMC chains each. The chains were run for 35,000,000 iterations, with the first 25,000,000 iterations discarded as burn-in, and sampled every 10,000 iterations after convergence, to produce a posterior sample of 1,000 predictive models and associated parameters.

### Bite force estimation

Using the predicted *A*_Phys_ I estimated bite force (*F*_Bite_) for 30 of the 33 fossil dinosaur species for which I predicted *A*_Phys_ through the PPM approach. I then compared those against *F*_Bite_ estimated for the 19 of the 20 fossil archosaurs based on measured *A*_Phys_ reconstructions included in the training set for the PPMs.

**Figure 4:**
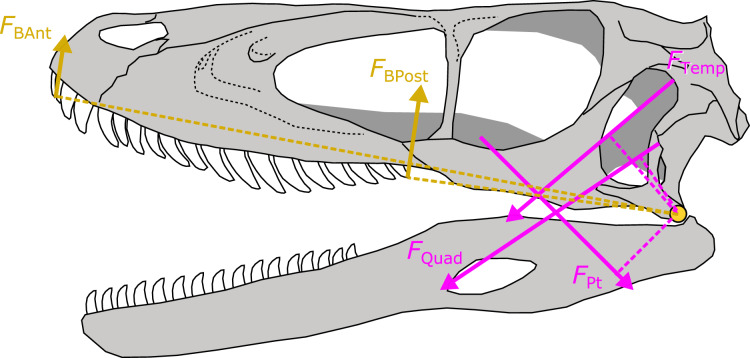
Schematic depiction of a static lever model to estimate bite force in extinct dinosaurs. Bite force (*F*_BAnt_ and *F*_BPost_) was estimated in extinct dinosaurs using a static lever model as shown on a skull and mandible reconstruction of *Deinonychus* (author’s own work). *F*_BAnt_, anterior bite force; *F*_BPost_, posterior bite force; *F*_Temp_, temporal group muscle force; *F*_Quad_, quadrate group muscle force; *F*_Pt_, pterygoid group muscle force.

For each of the 30 fossil species for which I predicted *A*_Phys_, I took the median value of the posterior predictive distribution for each muscle. Muscle force (*F*_Musc_) was then estimated for each muscle as the product of *A*_Phys_ and tetanic stress σ at 0.3 N/mm^2^ (or 300 kPa). The resulting *F*_Musc_ was then multiplied by the muscle moment arm to yield the torque of that muscle. I measured relevant moment arms for each muscle following the procedures developed in Sakamoto (2010) (Fig. 4). Muscle moments were summed and divided by the distance between the fulcrum (jaw joint) and bite point and multiplied by two to derive a bilateral *F*_Bite_. *F*_Bite_ was estimated for the anterior-most and posterior-most positions along the biting edge (tooth row or beak; Fig. 4).

*F*_Bite_ in fossil archosaurs that were included in the PPM training set (*N* = 19) were estimated in the same way as above but using *A*_Phys_ measured from reconstructed muscles as outline in Sakamoto, Ruta & Venditti (2019). I compared *F*_Bite_ at the posterior-most positions (maximum *F*_Bite_) between the two sets of fossil species.

## Results

### Prediction accuracies of PPMs

Prediction accuracies of the PPMs in the initial training set consisting of only extant species (*N* = 39) was at 87% for all three muscle groups. The prediction accuracies of the PPMs in predicting *A*_Phys_ for the 20 fossil species, as compared to their measured *A*_Phys_ were 25%, 45% and 35%, respectively for the mTemp, mQuad and mPt groups.

Prediction accuracies of the PPMs in the expanded training set including fossil species (*N* = 59) were at 95%, 93% and 90% for the mTemp, mQuad and mPt groups, respectively. Out of the 20 fossil species included in the training set, in only two species (*Plateosaurus engelhardti* and *Herrerasaurus ischigualastensis*) did the PPMs fail to predict the observed *A*_Phys_.

### Bite force estimation

**Figure 5:**
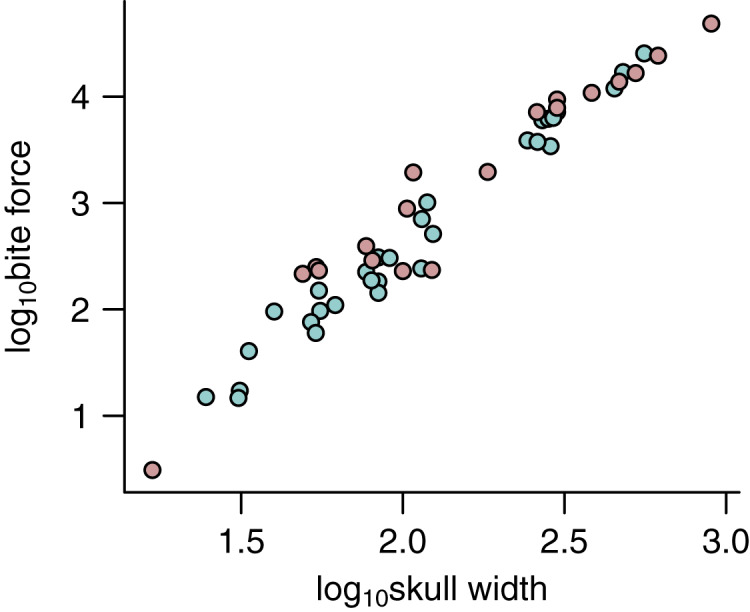
Relationship between bite force and skull width. The relationship between bite force and skull width is shown for estimates based on predicted *A*_Phys_ (light green) and those based on muscle reconstructions (pink).

*F*_Bite_ estimated for the 30 fossil species based on predicted *A*_Phys_ are shown in Table 1. Compared to *F*_Bite_ estimated from reconstructed *A*_Phys_ in the 19 fossil species, these 30 *F*_Bite_ values fall along the expected relationship between *F*_Bite_ and *W*_Sk_ (Fig. 5). Comparisons between closely related species of similar sizes reveal the accuracy in resulting *F*_Bite_ values (Table 1): *F*_Bite_ for *Deinonychus antirhoppus* (predicted *A*_Phys_) with *W*_Sk_ of 114.5 mm is 706N, while *F*_Bite_ for *Dromaeosaurus albertensis* (reconstructed *A*_Phys_) with *W*_Sk_ of 103 mm is 885N; *F*_Bite_ for *Carnotaurus sastrei* (predicted *A*_Phys_) with *W*_Sk_ of 300 mm is 7,172N while *F*_Bite_ for *Majungasaurus crenatissimus* (reconstructed *A*_Phys_) with *W*_Sk_ of 300 mm is 7,845N.

**Table 1:** Bite forces estimated in extinct dinosaurs using *A*_Phys_ values either predicted through the PPMs or from reconstructed muscles.

Taxon	*F*_BAnt_	*F*_BPost_	*W*_Sk_	*A*_Phys_
*Acrocanthosaurus atokensis*	8,266	16,984	480	Predicted
*Bambiraptor feinbergorum*	50	97	55.5	Predicted
*Baryonyx walkeri*	1,382	3,416	286	Predicted
*Carcharodontosaurus saharicus*	11,312	25,449	558	Predicted
*Carnotaurus sastrei*	3,392	7,172	300	Predicted
*Ceratosaurus nasicornis*	2,432	5,998	270	Predicted
*Citipati osmolskae*	202	225	77	Predicted
*Compsognathus longipes*	8	15	24.6	Predicted
*Confuciusornis sanctus*	12	17	31.3	Predicted
*Deinonychus antirrhopus*	298	706	114.5	Predicted
*Dilong paradoxus*	64	110	61.8	Predicted
*Eoraptor lunensis*	35	95	40	Predicted
*Gallimimus bullatus*	152	243	114	Predicted
*Garudimimus brevipes*	121	183	84	Predicted
*Guanlong wucaii*	268	512	124	Predicted
*Haplocheirus sollers*	46	76	52	Predicted
*Incisivosaurus gauthieri*	26	41	33.4	Predicted
*Monolophosaurus jiangi*	1,710	3,872	243	Predicted
*Nanotyrannus lancensis*	2,068	3,752	261	Predicted
*Nemegtomaia barsboldi*	236	308	84	Predicted
*Ornithomimus edmontonicus*	94	143	84	Predicted
*Shuvuuia deserti*	12	15	31	Predicted
*Sinornithosaurus millenii*	30	60	53.8	Predicted
*Spinosaurus aegyptiacus*	4,829	11,936	451	Predicted
*Struthiomimus altus*	108	187	80	Predicted
*Teratophoneus curriei*	2,812	6,188	282	Predicted
*Tsaagan mangas*	63	150	55	Predicted
*Velociraptor mongoliensis*	131	304	91	Predicted
*Yangchuanosaurus shangyouensis*	3,212	6,312	292	Predicted
*Zupaysaurus rougieri*	325	1,012	119	Predicted
*Allosaurus fragilis*	4,440	9,389	300	Reconstructed
*Archaeopteryx lithographica*	2	3	16.8	Reconstructed
*Coelophysis bauri*	72	289	74	Reconstructed
*Coelophysis rhodesiensis*	99	393	77	Reconstructed
*Daspletosaurus torosus*	8,385	16,641	525	Reconstructed
*Dromaeosaurus albertensis*	443	885	103	Reconstructed
*Erlikosaurus andrewsi*	118	229	100	Reconstructed
*Euparkeria capensis*	86	216	49	Reconstructed
*Gorgosaurus libratus*	6,418	13,817	467	Reconstructed
*Herrerasaurus ischigualastensis*	678	1,937	107.7	Reconstructed
*Lesothosaurus diagnosticus*	99	250	54	Reconstructed
*Majungasaurus crenatissimus*	3,140	7,845	300	Reconstructed
*Ornithosuchus woodwardi*	2,910	7,146	260	Reconstructed
*Parasuchus hislopi*	450	1,958	183	Reconstructed
*Plateosaurus engelhardti*	82	235	123	Reconstructed
*Riojasuchus tenuisceps*	109	232	55	Reconstructed
*Sinraptor dongi*	5,064	10,845	384	Reconstructed
*Tarbosaurus bataar*	13,298	24,253	616	Reconstructed
*Tyrannosaurus rex*	25,418	48,505	900	Reconstructed

**Note:**

*F*_BAnt_, anterior bite force; *F*_BPost_, posterior bite force; *W*_Sk_, skull width; and *A*_Phys_, physiological cross-sectional area.

## Discussion

The analyses presented here largely demonstrate two interesting features of predicting *A*_Phys_ in extinct species. First, the addition of *A*_Phys_ values for extinct species (measured from reconstructed muscles) in the training set drastically improved prediction accuracy of extinct species: compare 25–45% prediction accuracy using the extant-only PPM with 90% prediction accuracy (18/20 species) using the PPM that includes fossil species. The most likely cause of this improvement is increased sample size, from *N* = 39 to *N* = 59. It is well known and demonstrated through simulations that evolutionary parameters, such as Pagel’s λ (Pagel, 1997) lose statistical power with smaller sample size, with a marked reduction at approximately *N* < 50 (Freckleton, Harvey & Pagel, 2002; Sakamoto & Venditti, 2018). As the PPM approach taken here is also based on the Brownian motion model of phenotypic evolution, it has similar statistical properties to estimating λ, and would most likely encounter similar effects of sample size. Thus, increasing sample size to *N* = 59, which is just above this threshold of *N* = 50 previously suggested through both simulated (Freckleton, Harvey & Pagel, 2002) and empirical (Sakamoto & Venditti, 2018) cases, is likely the underlying cause for the improvement in prediction accuracy. Indeed, a similar recent study, which demonstrated that *F*_Bite_ estimated in extinct species are in line with those expected for animals of similar sizes, used a PPM based entirely on extant species but had a sample size of *N* = 188 (Sakamoto, 2021).

It is also possible however that the act of including fossil estimates in and of itself does have some positive effect on improving prediction accuracy. It has been shown before that inclusion of extinct tips in phylogenetic comparative analyses preserved phylogenetic signal λ (Pagel, 1997) in rates of phenotypic evolution deeper in the tree, while that of ultrametric trees degraded rapidly (Sakamoto & Venditti, 2018) – one interpretation is that subsequent evolution ‘overwrote’ signals from deeper in the tree when only data from extant taxa are modelled, but including fossil data deeper in the tree adds this information into the model. Thus, data associated with extinct tips that are deeper in time likely improves parameter estimation in phylogenetic comparative models. The oldest species in my dataset are approximately 250 million years old and comparatively close in time to the root of the tree and may contribute to this type of effect on evolutionary parameter (*e.g*., Brownian variance) estimation and the resulting posterior distribution of predictive models.

Crucially, as *A*_Phys_ in dinosaurs are generally much larger than those in most of the extant species in this dataset (Fig. 5), predicting for dinosaurs from PPMs based on the extant-only training set (*N* = 39) is effectively extrapolating far beyond the range of the data.

A note of caution however is that low prediction accuracy of *A*_Phys_ in extinct species using the extant-only training set may also be indicative of uncertainties related to muscle reconstructions based on skull geometries (Bates et al., 2021). However, high precision accuracy in the LOOCV of the *N* = 59 training set indicate that variation within *A*_Phys_ from muscle reconstructions in extinct species are within expected range of variance given phylogeny and Brownian motion (Sakamoto, 2021). Crucially, the objective of this study is to develop a method to predict *A*_Phys_ in extinct species from skull geometries that are within the same range of accuracy as those measured from reconstructed muscles, not to accurately predict real in-life *A*_Phys_ values as a substitute of muscle reconstruction – *i.e*., this method is to augment gaps in muscle reconstructed *A*_Phys_ data, not to replace muscle reconstructions entirely. Increased prediction accuracy by expanding the training set (*N* = 59) to include *A*_Phys_ estimates for 20 extinct species fulfils this purpose.

Second, the power of simple linear morphometrics (*e.g*., *W*_Sk_) in predicting functionally important parameters is not to be taken lightly. The PPMs developed here are based only on *W*_Sk_ but is demonstrated to have prediction accuracy upwards of 95% depending on the muscle group. The fact that *W*_Sk_ is tightly correlated with *F*_Bite_ across multiple groups of vertebrates (Sakamoto, 2021; Herrel et al., 2005b) is consistent with these results. *W*_Sk_ is also tightly linked with body size, often scaling isometrically, making it the ideal predictor in PPMs for its ability to ground the model to a theoretical scaling framework, *e.g*., expected scaling exponent between area and length (Fig. 2). Simple metrics are also readily available across a wide taxonomic sample and can be collected from literature and osteological specimens, including fossils. PPMs based on such simple predictors are thus more versatile and robust.

### Bite force estimates in extinct archosaurs

Using the *A*_Phys_ predicted from the PPMs, I estimated *F*_Bite_ in several extinct archosaurs. These values can be regarded as reasonably reliable estimates of true *F*_Bite_ in these extinct animals, given scaling relation with *W*_Sk_ and phylogeny. This owes to the fact that they are highly congruent with *F*_Bite_ estimates based on *A*_Phys_ measured from reconstructed muscles, which themselves have been demonstrated to be reasonably reliable estimates of *F*_Bite_ given size and phylogeny (Sakamoto, 2021). There are still some notable discrepancies between *F*_Bite_ estimated here with published values, namely in oviraptorosaurs (Meade & Ma, 2022) and ornithomimosaurs (Cuff & Rayfield, 2015), but also in *Deinonychus* (Gignac et al., 2010). My estimated *F*_Bite_ for oviraptorosaurs are under-estimates of published figures (*Citipati osmolskae*, 202–225N *vs* 349.3–499.0N (Meade & Ma, 2022); *Incisivosaurus gauthieri*, 26–41N *vs* 53–82.5N (Meade & Ma, 2022)) while those for ornithomimosaurs are over-estimates (*Garudimimus*, 121N at the tip *vs* 19N (Cuff & Rayfield, 2015); *Ornithomimus*, 121N *vs* 22N (Cuff & Rayfield, 2015); *Struthiomimus*, 108N *vs* 57.6N (Cuff & Rayfield, 2015)). The under-estimation of oviraptorosaurs is within the same order of magnitude (×10^1^) and thus trivial in a comparative context (Sakamoto, 2021). On the other hand, the over-estimation of ornithomimosaurs is one order of magnitude (×10^2^ *vs* ×10^1^). The likely source of this discrepancy lies in the fact that neither oviraptorosaurs nor ornithomimosaurs were included in my *N* = 59 training set, thus making the predicted *A*_Phys_ reflecting values that are more typical of closely related theropod dinosaurs (Fig. 4; Table 1) as well as regressing to the mean of the training set (as is typical for regression). Future work expanding on the taxonomic sampling of the training set will undoubtedly improve prediction accuracy in specific taxa, especially in clades with unique dietary adaptations such as oviraptorosaurs (Sakamoto, 2010; Meade & Ma, 2022) or ornithomimosaurs (Cuff & Rayfield, 2015).

The case of discrepancy in *F*_Bite_ estimates for *Deinonychus* between this study and that by Gignac et al. (2010) warrants some special attention. Gignac et al. (2010) predicted the forces necessary to puncture bone to the depth observed in *Tenontosaurus* bones (Gignac et al., 2010; Erickson et al., 1996) and estimated the maximum *F*_Bite_ at the posterior-most biting position for *Deinonychus* at 8,200N, which is an order of magnitude higher than that estimated here at 706N (Table 1). My estimated *F*_Bite_ of 706N is in line with those of a similarly sized dromaeosaur *Dromaeosaurus* at 885N (*W*_Sk_ = 103 mm), and a similarly sized basal saurischian *Herrerasaurus* at 1,937N (*W*_Sk_ = 108 mm) (Table 1), but also extant diapsids with similar skull widths, *Paleosuchus trigonatus* at 1,082N (*W*_Sk_ = 121 mm), and *Alligator sinensis* at 1,084N (*W*_Sk_ = 122 mm) (Sakamoto, Ruta & Venditti, 2019). Interestingly the same can be said when the comparison is extended to extant carnivores with similar skull widths, *Ursus thibetanus* at 871N (*W*_Sk_ = 111 mm), *Neofelis nebulosa* at 1,296N (*W*_Sk_ = 119 mm), *N. diardi* at 1,117N (*W*_Sk_ = 115 mm), and *Sarcophilus harrisii* at 682N (*W*_Sk_ = 112 mm) (Sakamoto, Ruta & Venditti, 2019). It is important to note that forces necessary to puncture substrate are not confined to muscle-driven biting and may very well be the product of more aggressive kinetic feeding behaviours involving the whole head and neck (D’Amore & Blumenschine, 2009; Snively et al., 2013; Torices et al., 2018). This is supported by the observation that this bite mark in question was matched to a premaxillary tooth (Gignac et al., 2010), meaning that a long-snouted animal would have had to be capable of generating *F*_Bite_ of 3,000–4,000N (Gignac et al., 2010) at the tip of its snout through muscle generated biting, which is not congruent with the available data in comparably-sized amniotes (Sakamoto, Ruta & Venditti, 2019). Given its congruence with a similarly sized dromaeosaur *Dromaeosaurus* as well as other similarly sized amniotes, there is strong evidence to suggest that my *F*_Bite_ estimate for *Deinonychus* is reasonably accurate for an animal of its size.

Crucially, a comparison of *F*_Bite_ based on predicted *A*_Phys_ (*N* = 30) and *F*_Bite_ based on reconstructed *A*_Phys_ (*N* = 19) across the extinct non-avian theropods (Fig. 5; Table 1) generally show substantial overlap without any signs of sytemic biases (Fig. 5). This demonstrates that *F*_Bite_ estimates based on predicted *A*_Phys_ are neither systemically over- or under-estimates compared to *F*_Bite_ based on reconstructed *A*_Phys_. Thus, PPMs are a useful approach to expand on reliable *F*_Bite_ data based on simple metrics and phylogeny to augment those based on reconstructed muscles. Importantly, this approach allows estimation of *A*_Phys_ and *F*_Bite_ in taxa (both extinct and extant) where muscle reconstruction is not feasible or possible for any number of reasons.

Discussion of *F*_Bite_ for individual species of interest are then valid and worth considering. Of note is that the large-bodied carnivorous dinosaurs, *Carcharodontosaurus saharicus* and *Acrocanthosaurus atokensis*, both reaching the size range of *Tyrannosaurus rex*, have *F*_Bite_ that are substantially lower than the latter, at 16,984 and 25,449N respectively, compared to 48,505N of *T. rex*. *Carcharodontosaurus* is approximately the same size as *T. rex* but is here shown to have had *F*_Bite_ that is approximately half of the latter. *Carcharodontosaurus* is typical in build and skull proportion for a theropod dinosaur, so the fact that its *F*_Bite_ was only half of that of *T. rex* is more likely a reflection of just how unique *T. rex* may have been compared to other theropods of similar sizes. *Tyrannosaurus* had robust conical-shaped teeth and multiple adaptations in the skull that allowed it to withstand immense forces (Cost et al., 2019; Rayfield, 2005; Snively, Henderson & Phillips, 2006). Multiple lines of evidence also point to habitual bone-crushing and -consumption in *T. rex* (Gignac & Erickson, 2017; Erickson et al., 1996; Erickson & Olson, 1996). These support the hypothesis that *T. rex* had at least a partial osteophagous diet, an ecology that was likely different from other theropods.

A similarly, large-bodied carnivorous dinosaur, *Spinosaurus aegyptiacus*, is here tentatively predicted to have had *F*_Bite_ at just under 12,000N, roughly in the same range as *Sinraptor* (10,845N), *Gorgosaurus* (13,817N), and *Daspletosaurus* (16,641N), all substantially smaller theropods. With the caveat that *W*_Sk_ for *Spinosaurus* was simply scaled up from the skull-width ratio of *Suchomimus* (Sereno et al., 1998), I offer additional support for this taxon to have had unique feeding habits for a theropod of its size. *Spinosaurus* shows adaptations in the craniomandibular morpho-functional complexes that are advantageous for generating relatively faster shutting speeds with less muscle input force (higher displacement advantage) at the expense of *F*_Bite_ (lower mechanical advantage) (Sakamoto, 2010). This would be congruent with a feeding mode relying on fast-snapping jaws rather than slow crushing bites, which is commonly observed in species with semi-aquatic feeding habits, including herons and egrets (Hone & Holtz, 2021).

### Wider implications

The phylogenetic predictive framework I present here enhances collection of trait data that may be difficult to obtain across a wide taxonomic sample. Various *in vivo* measurements (*e.g*., bite force, muscle architecture) are obviously impossible to collect in extinct taxa, but they may also be difficult to obtain in many extant taxa, especially for those that are exotic, enigmatic, endangered, or dangerous. PPM then allows for predictions of trait data in such taxa provided that more readily available predictor variables (*e.g*., external physical traits) can be measured for them.

While the case study presented here focused on predicting muscle parameters, the PPM approach is not restricted to soft tissue predictions. The response variable Y of interest can be any single continuously varying trait as long as it exhibits significant relationships with a set of predictor variables X_i_ in taxa for which both Y and X_i_ can be measured. Examples include (but not precluded to): predicting body mass from skeletal measurements; predicting skeletal structural strength index from skeletal variables; or predicting metabolism from body mass.

Similarly, this framework is not restricted to any taxonomic group or scope. The taxonomic group of interest can be of any nature and breadth as long as a phylogeny exists that includes the taxa for which the Y variable is to be predicted.

A key feature of phylogenetic regression that makes the PPM framework extremely versatile is that it is extremely robust to uncertainties in terms of phylogenetic relations and branch lengths (Stone, 2011). This means that even a phylogeny with high levels of uncertainties (especially branch lengths for fossil trees) can be used effectively in a PPM framework to predict Y variables of interest. Nevertheless, modern approaches to branch scaling such as the tip-dating approach (Drummond & Stadler, 2016) that dates the divergences and tips simultaneously with topology inference (Gavryushkina et al., 2017; Stadler et al., 2018) can return a set of branch lengths that are guided by data and likelihood (*i.e*., probabilistic estimates), but these should be conducted using phylogenetic/cladistic data matrices (*e.g*., molecular or morphological characters), appropriate model of evolution, and tree prior, rather than on a fixed topology with no character data as is increasingly being used (Godoy et al., 2021; Gearty & Payne, 2020). The parameters associated with the latter are likely returning the priors as there are no data informing the likelihood, making the set of scaled branches no better than sampling randomly, but more investigation is needed.

It is important to note however that when considering Y and taxonomic sampling, the Y variable of interest should be broadly homologous across the phylogeny used, especially if the phylogenetic coverage is broad, spanning several higher order taxonomic groups, *e.g*., synapsids and diapsids. For instance, jaw adductor muscles of mammals (*e.g*., temporal and masseter muscles) are not directly comparable to those of diapsids (*e.g*., the mTemp, mQuad and mPt groups used here), but they are homologous within Mammalia across mammalian orders. On the other hand, biomechanical performance measures such as bite force is homologous across a very large portion of the tree of life, *e.g*., across vertebrates. The consideration here then would be the homology between the predictor variables across vertebrate clades with vastly diverse skull anatomy – *e.g*., skull measurements across various fish clades may not be precisely homologous, which is exemplified when the comparisons extend to amniotes.

There is no multi-response (multivariate regression) implementation as of date, but this is true for phylogenetic regression in general. This means that applications such as predicting morphospace coordinates would need to be conducted on individual shape (*e.g*., principal components) axis separately.

## Conclusions

Here, I present a phylogenetic predictive modelling framework to predict soft tissue parameters (*A*_Phys_) in extinct species from an osteological predictor variable (*W*_Sk_). Predicted parameters are reasonably accurate given the known scaling relationship between the muscle parameter and predictor variable and phylogeny. Downstream biomechanical modelling yields performance metrics (*F*_Bite_) that are in line with previous estimates based on muscle parameters from reconstructed muscles. Thus, phylogenetic predictive modelling provides a powerful means to predict soft tissue parameters for biomechanical modelling in extinct species from simple osteological predictor variables.

## Supplemental Information

10.7717/peerj.13731/supp-1 Supplemental Information 1 Raw data – *A*_Phys_ in extant reptiles.

10.7717/peerj.13731/supp-2 Supplemental Information 2 Raw data – *A*_Phys_ in extinct archosaurs.

10.7717/peerj.13731/supp-3 Supplemental Information 3 APhys data.

10.7717/peerj.13731/supp-4 Supplemental Information 4 Phylogenetic tree.

10.7717/peerj.13731/supp-5 Supplemental Information 5 LOOCV results N39 model.

10.7717/peerj.13731/supp-6 Supplemental Information 6 LOOCV results N59 model.

10.7717/peerj.13731/supp-7 Supplemental Information 7 Predicted APhys in fossil taxa.

10.7717/peerj.13731/supp-8 Supplemental Information 8 Estimated bite forces for extinct taxa. Bite forces estimated using either *A*_Phys_ predicted from the phylogenetic predictive models or from reconstructed muscles

10.7717/peerj.13731/supp-9 Supplemental Information 9 Estimated bite force in theropod dinosaurs.

## Additional Information and Declarations

### Data Availability

The following information was supplied regarding data availability: The data is available at Open Science Framework: Sakamoto, Manabu. 2022. “Estimating Bite Force in Extinct Dinosaurs Using Phylogenetically Predicted Physiological Cross-Sectional Areas of Jaw Adductor Muscles.” OSF. April 23. DOI 10.17605/OSF.IO/VXD59.
